# Patients with young-onset dementia in an older people's mental health service

**DOI:** 10.1192/bjb.2020.89

**Published:** 2021-04

**Authors:** Michael Yeung, Katherine MacFarland, Vincent Mlilo, Nathan Dean, Benjamin R. Underwood

**Affiliations:** 1 University of Cambridge; 2 North Middlesex University Hospital NHS Trust; 3 Cambridgeshire and Peterborough NHS Foundation Trust

**Keywords:** Dementia, depressive disorders, YoD, young-onset dementia, service evaluation

## Abstract

**Aims and method:**

Currently, no separate service exists for patients with young-onset dementia in Cambridgeshire. These patients are managed together with late-onset dementia patients within old age psychiatry services. To inform service design, we sought to characterise young-onset dementia patients in our population. We first analysed service-level data and supplemented this with a detailed case review of 90 patients.

**Results:**

Young-onset dementia remains a relatively rare condition. Only a small proportion of those referred for assessment receive a diagnosis of dementia. Data collected on presenting complaints, comorbidities, medication and Health of the Nation Outcome Scales scores associated young-onset dementia with a greater incidence of depression than late-onset dementia. Outcomes in the two groups did not appear to differ.

**Clinical implications:**

The data presented here do not suggest a need to create a separate service. Practitioners should be aware of the increased incidence of depression observed in this group.

Dementia is a growing national and international problem with associated personal and societal costs. For example, in Cambridgeshire, the number of individuals with dementia is predicted to increase by 86% from 8600 in 2016 to 16 110 by 2031.^1^ Of specific interest are those who develop dementia at a young age. Young-onset dementia (YoD) is defined as a diagnosis prior to the age of 65, a cut-off based on the previous retirement age and not on any biological underpinning.^2^ Both YoD and late-onset dementia (LoD) represent heterogeneous groups of patients, which differ from each other in various features besides age. Although the incidence of dementia increases with age, those who develop dementia at a young age have a different profile of diagnosis compared with older people. A greater proportion of YoD patients suffer from frontotemporal lobar degeneration, and they may experience delays in diagnosis.^3,4^ Furthermore, studies have shown a higher neuropsychiatric symptom burden and greater carer stress.^5^ These differences have prompted discussions regarding the need for a separate specialist service for those with YoD.^6^

Currently, YoD patients are treated together with LoD patients within old age psychiatry services. Referrals are made to the same memory clinic, where patients are assessed by a consultant specialising in old age psychiatry, or by a trainee or middle-grade doctor under their supervision. Over the past 4 years, older people's mental health (OPMH) services have undergone a radical transformation in Cambridgeshire.^7^ In order to inform future service design, we therefore sought to evaluate our patients with established YoD and patients under 65 referred for memory assessment, in comparison with those with LoD.

## Methods

This project was conducted as a service evaluation with the relevant internal trust approvals from Cambridgeshire and Peterborough NHS Foundation Trust. The trust covers a population of ~1 000 000 people, of whom >165 000 are over the age of 65. The trust provides the countywide memory assessment service, which receives ~2000 referrals per year. Other condition-specific services exist (for example, for Huntington's disease) and sit within the neurology department at the local acute trust.

Referral data are routinely collected by the trust. Health of the Nation Outcome Scales (HoNOS) data were collected as previously described.^8^ We manually reviewed a sample of 90 electronic patient records. This was divided into data for 30 consecutive patients with an established diagnosis of YoD, 30 consecutive patients with a diagnosis of LoD, and 30 consecutive patients under the age of 65 who had been referred to the memory clinic for diagnostic assessment. For each patient, we searched their records to identify the following: presenting complaint, diagnosis, presence of comorbidities, time from symptom onset to diagnosis, current medication, and scores on the Addenbrooke's Cognitive Examination (ACE) and HoNOS. We attempted to minimise the possibility of interrater variability by looking for specific data and using the same source data in the records (the core assessment). The date of symptom onset was determined from the information provided in the core assessment, which is a mandatory form that includes a detailed patient history, completed by a clinician for each patient. Data for individuals identified with dementia in the young referral group were combined with data from the YoD group during statistical analysis in order to increase statistical power. χ^2^-test, unpaired t-test and Fisher's exact test were used as appropriate for statistical analysis of the data (see supplementary material, available online at https://doi.org/10.1192/bjb.2020.89). *P*-values were not adjusted for multiple testing.

## Results

**Fig. 1:**
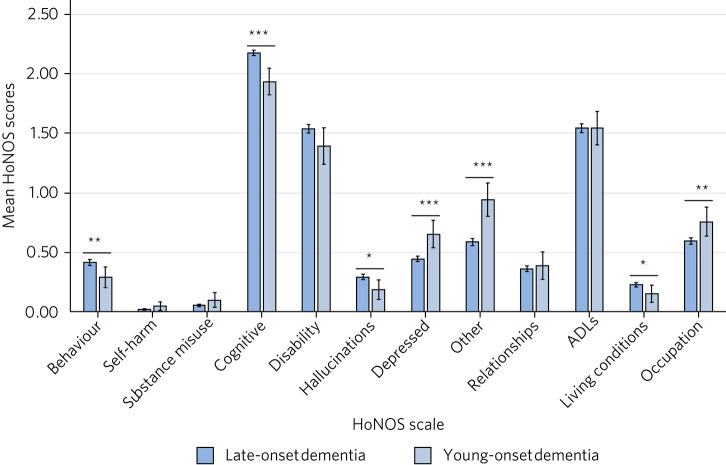
Bar chart showing mean scores across HoNOS scales for late- and young-onset dementia on presentation. Late-onset dementia: *N* = 3553. Young-onset dementia: *N* = 173. *P < 0.05, **P < 0.01, ***P < 0.001. ADLs, activities of daily living.

We began by looking at high-level data regarding case-load, referrals and outcomes of people with YoD. In March of 2020, the trust had a total of 5818 registered patients with a diagnosis of dementia, i.e. patients on our electronic patient record but not necessarily currently receiving a service. Of these, 135 (2.3%) were under the age of 65. Of 7473 individuals referred for memory assessment between 2016 and 2020, 210 were under the age of 65, corresponding to 2.8% of all referrals. We re-analysed a large data set of HoNOS scores which we had previously reported, in order to investigate differences in presentation and outcomes between YoD and LoD. This comprised data for 173 patients with YoD and 3553 patients with LoD,^8^ representing the subset of total referrals that had a HoNOS score on admission and discharge. Using this methodology, we found significant differences in HoNOS scores on entry to the services (data not previously presented), with higher scores on the behaviour (*P* = 0.01), cognition (*P* < 0.001), hallucinations (*P* = 0.01) and living conditions (*P* = 0.04) domains in the LoD group; and higher scores for depression (*P* < 0.001), occupation (*P* = 0.01) and ‘other’ (*P* < 0.001) in the YoD group (Fig. 1). There were no significant differences in the other domains. We have previously published data looking at outcomes based on HoNOS scores for both YoD and LoD. Both groups improved in all domains except cognition, disability and activities of daily living. Although some of these changes did not reach statistical significance in the YoD group, this is likely to reflect the lower numbers in that group.^8^

**Fig. 2:**
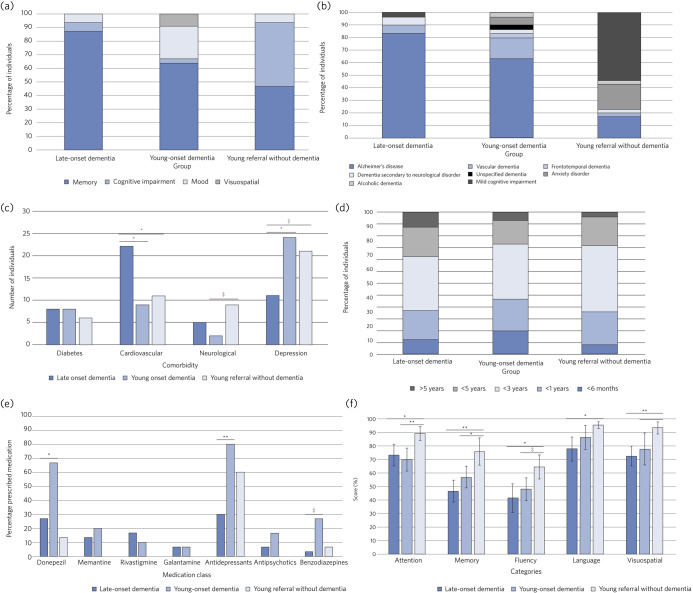
(a) Bar chart showing presenting complaints in each group. (b) Bar chart showing proportions of different diagnoses in each group. (c) Bar chart showing number of individuals associated with different comorbidities in each group. (d) Bar chart showing time from symptom onset to diagnosis in each group. (e) Bar chart showing percentage of patients prescribed different medication classes in each group. (f) Bar chart showing mean ACE scores in each category for each group. *P < 0.05, **P < 0.01, ***P < 0.001.

In order to conduct a more comprehensive investigation into the differences between our YoD and LoD patients, we undertook a detailed case-note review of 30 cases from each group. The demographic details are given in Table 1. We found significant differences between the YoD and LoD groups in terms of comorbidities (*P* = 0.002 for both cardiovascular and depressive) and medication (*P* = 0.0003 for antidepressants, *P* = 0.004 for donepezil and *P* = 0.03 for benzodiazepines). Specifically, we saw a higher incidence of mood as a presentation (Fig. 2a), a greater variety of diagnoses with less Alzheimer's disease (Fig. 2b), fewer cardiovascular and more depressive comorbidities (Fig. 2c), a generally shorter time from symptom onset to diagnosis (40% diagnosed in less than 1 year for YoD compared with 30% for LoD, although these differences were not significant, nor was the difference in mean time to diagnosis of 27 *v.* 28 months for YoD *v.* LoD, respectively, Fig. 2d), and more treatment with donepezil, antidepressants and sedative medication in the YoD group (Fig. 2e). We found no statistical difference in total ACE scores or subscores between YoD and LoD (Fig. 2f). Two patients in the YoD group who initially received a diagnosis of dementia subsequently had that diagnosis removed, as it became clear that their symptoms were a result of other psychiatric disorders.

**Table 1:** Sample demographics of late-onset dementia, young-onset dementia and young referrals without dementia groups

	Late-onset dementia	Young-onset dementia	Young referral without dementia
Mean age at diagnosis (s.d.)^a^	83.4 (±6.8)	56.4 (±5.4)	55.8 (±7.8)
Minimum age^a^	72	45	35
Maximum age^a^	97	64	64
Male	14	16	17
Female	16	14	13
Mean age of males^a^	82.4	56.0	54.2
Mean age of females^a^	84.3	56.9	57.6

a. Age in years.

We also evaluated 30 cases of patients under 65 years of age referred for memory assessment. When comparing patients referred to our service under the age of 65 with those in the same age group who had received a diagnosis of dementia, we found a number of differences. First, despite the similar average ages of the two groups, there was a greater range in those referred, with one individual as young as 35 years of age who was not subsequently diagnosed with dementia. For total and subscores on the ACE and cognitive scores on HoNOS, those referred scored significantly higher (mean ± s.d. for total ACE: 84 ± 11 *v.* 69 ± 17), were less likely to have difficulties with memory as a presenting complaint and were more likely to have neurological comorbidities. The prevalence of diagnosis of dementia in this group was low (27%). No other significant differences were found.

## Discussion

The absolute numbers of patients with YoD referred or managed are low, representing just over 2% of referrals and case-load. This is lower than the figure found in the national memory service audit for referral (7%) and at the lower end of the range (0–22%).^9^ It is also lower than many estimates of the prevalence of dementia in this age group.^10^ For example, there are an estimated 210 individuals with YoD in Cambridgeshire, but only 135 (64%) of these are currently known to the trust. This difference might be explained by many of the estimates of prevalence being based on epidemiological data, meaning there are likely to be individuals who have the condition but have not yet been diagnosed. Given the increased prevalence of certain conditions leading to dementia in this group, including Huntington's disease and frontotemporal dementia, it is also possible that they are looked after in the relevant specialist neurological clinic rather than by generic mental health services. This may also explain the low percentage in terms of referrals, if doctors in primary care are preferentially referring young patients to neurology rather than psychiatry for assessment.

For the cohort under 65 who were referred for diagnostic assessment, only eight out of 30 (27%) received a dementia diagnosis. This is much lower than national figures for generic memory clinics or those from our previously published audits of our own service, where 60–70% of those referred received a diagnosis of dementia.^11^ However, this supports previous data from the London memory audit services, where only 15% of those referred under 65 received a dementia diagnosis.^12^ Instead, the majority in this group were diagnosed with mild cognitive impairment or other psychiatric disorders. Our analysis of HoNOS scores of patients on entry to the service did not broadly support the idea that YoD patients, at least at presentation, suffer from more neuropsychiatric disturbance. However, the evidence presented here based on HoNOS scores, presenting complaint, comorbidity and medication suggests that this group is associated with a greater burden of depression.

The association of depression with LoD has been well documented, with large-scale studies suggesting that depression is both a prodromal symptom of and a risk factor for dementia, while dementia is itself a risk factor for depression.^13^ A recent meta-analysis reported a prevalence of depression of 25% in those with LoD.^14^ This is in contrast to studies of YoD, where depression was found in 66% of individuals.^15^ Although assessing depression is difficult in those with dementia, and criteria differ among studies, our data support the idea that patients with YoD may suffer more from depression. From a diagnostic perspective, this is potentially an important consideration when seeing patients in later mid-life who are suffering from mood disorder, which may be comorbid with or indeed be a presentation of YoD. This is also important in terms of management, as depression is both under-diagnosed and under-treated in this population, which may negatively affect prognosis.^15^

However, our study did not support previous suggestions of a delayed diagnosis in those with YoD, although we acknowledge the small sample size and difficulty people experience in precisely recalling when the onset of an insidious condition might have been. One possible explanation is that a greater proportion of those with Alzheimer's disease are seen in our service, and fewer of those with rarer and therefore more difficult to diagnose dementias such as frontotemporal lobar degeneration, who may instead be referred to a different service such as neurology.^4^ Another possibility is that our patient population may not be representative of studies in other populations. In terms of socioeconomic background, Cambridgeshire and Peterborough benefit from a slightly higher than average employment rate (78.5% in those aged 16–64 compared with 76% nationwide), as well as a higher percentage working in professional occupations (25.1% compared with 21.5% nationwide).^16^ A better socioeconomic background may provide the freedom for individuals to access health services at an earlier stage of the disease, which may be more difficult for those from less advantaged backgrounds. However, this would not explain why a difference between YoD and LoD groups was seen, as there is no reason to expect a greater effect in one group over another, and we are unaware of any facility for private dementia assessments available in the county.

We were interested to see that two cases initially diagnosed as YoD were subsequently reclassified with a diagnosis of another psychiatric disorder. This is a rare event in LoD and may reflect the lower pre-test probability of dementia in younger people, as well as the frequency of cognitive impairment in other psychiatric conditions. We were reassured that outcomes between patients with YoD and LoD did not appear to differ significantly.

In Cambridgeshire, we have used these data to inform the design of our services for YoD patients. We do not have a specialist YoD team. The low number of patients spread across more than 1300 square miles of a predominantly rural county makes having a specialist team practically challenging. A separate service dealing with YoD would be small by its nature and therefore not robust to any challenge such as staff sickness. Similarly, we do not have specialist clinics within the trust for those with YoD. The data suggesting a high level of psychiatric morbidity in this group make assessment by a consultant psychiatrist appropriate, and we have close links with local neurologists, including cognitive neurologists, for second opinions on cases which might represent Huntington's disease or unusual tau- or synucleinopathies. We do recognise the differences we see in our population and more broadly in the literature in those with YoD and the specific challenges this group can face. Our solution for their management has been to identify an advanced practitioner in each of our community memory teams who leads for YoD. This allows that practitioner to acquire expertise and experience in this area, forming part of a specialist professional group, as well as being part of a larger, multidisciplinary, clinical dementia service, which means the service offer is robust. We have also forged links with our local acute trust to ensure that patients seen in other related services, such as neurology, who receive a diagnosis of dementia are referred to our trust for post-diagnostic support and follow-up. One significant weakness in the data presented here was the lack of direct patient feedback. We do routinely collect quantitative and qualitative data from patients and caregivers. However, owing to incomplete returns from an already small group and not differentiating respondents in terms of age, this remains a significant gap in our knowledge. We will seek to address this in time with a targeted and more detailed assessment of patient experience, as well as detailed exploration of patients’ and carers’ ideas for service development.

In summary, our data suggest that patients with YoD form a small minority of our OPMH dementia work, and that the size of the population would make the creation of specialist teams difficult when operating over a large area. Young patients referred for assessment were less likely to receive a dementia diagnosis than older patients and were more likely to have psychiatric comorbidities. For those with YoD, their presenting complaint, medication, comorbidity and HoNOS scores all suggested a greater burden of depression. This information has helped us to inform and adapt our generic memory services to ensure a robust response led by staff experienced in this condition.

## About the authors

**Michael Yeung** is a medical student at the University of Cambridge, Cambridge, UK. **Katherine MacFarland** is a junior doctor at North Middlesex University Hospital NHS Trust, London, UK. **Vincent Mlilo** is a clinical research nurse in the Department of Clinical Neurosciences at the University of Cambridge, based in the Windsor Research Unit, which delivers clinical trials in dementia and mild cognitive impairment for patients in the NHS, and at Cambridgeshire and Peterborough NHS Foundation Trust, Fulbourn, UK. **Nathan Dean** is a medical student at the School of Clinical Medicine, Jesus College, University of Cambridge, Cambridge, UK. **Benjamin R. Underwood** is a consultant psychiatrist at the Windsor Unit, Cambridgeshire and Peterborough NHS Foundation Trust, Cambridge, UK.
