# Supplementary material for: Patients with young-onset dementia in an older people's mental health service
Source: BJPsych Bull. 2021 Apr;45(2):81–6. doi: 10.1192/bjb.2020.89 (PMC8111944; doi:10.1192/bjb.2020.89)

Supplemental data: statistical analysis

Abbreviations

LOD = late onset dementia

YOD = young onset dementia

YRWF = young referral without dementia

STDEV = standard deviation

CI = confidence intervals

| **HoNOS data** |
| --- |

|  | Behaviour | Self-harm | Substance abuse | Cognitive | Disability | Hallucinations | Depressed | Other | Relationships | ADL | Living conditions | Occupation |
| --- | --- | --- | --- | --- | --- | --- | --- | --- | --- | --- | --- | --- |
| LOD | 0.41 | 0.02 | 0.05 | 2.18 | 1.54 | 0.29 | 0.44 | 0.59 | 0.36 | 1.54 | 0.23 | 0.59 |
| YOD | 0.29 | 0.05 | 0.10 | 1.94 | 1.39 | 0.18 | 0.65 | 0.94 | 0.39 | 1.54 | 0.15 | 0.76 |
| STDEV LOD | 0.76 | 0.17 | 0.31 | 0.69 | 1.08 | 0.71 | 0.68 | 0.91 | 0.71 | 1.13 | 0.56 | 0.82 |
| STDEV YOD | 0.56 | 0.21 | 0.32 | 0.73 | 1.01 | 0.53 | 0.77 | 0.93 | 0.76 | 0.94 | 0.48 | 0.79 |
| CI LOD | 0.02 | 0.01 | 0.01 | 0.02 | 0.04 | 0.02 | 0.02 | 0.03 | 0.02 | 0.04 | 0.02 | 0.03 |
| CI LOD | 0.08 | 0.03 | 0.05 | 0.11 | 0.15 | 0.08 | 0.11 | 0.14 | 0.11 | 0.14 | 0.07 | 0.12 |
| p-value | 0.01 | 0.11 | 0.12 | <0.001 | 0.06 | 0.01 | <0.001 | <0.001 | 0.62 | 1.00 | 0.04 | 0.01 |

**ACE data**

|  | Attention | Memory | Fluency | Language | Visuospatial |
| --- | --- | --- | --- | --- | --- |
| LOD | 73.06 | 46.35 | 41.43 | 77.69 | 72.19 |
| YOD | 69.72 | 56.73 | 47.86 | 85.96 | 77.50 |
| YRWD | 88.89 | 75.71 | 64.29 | 95.14 | 93.09 |
| STDEV LOD | 18.58 | 18.74 | 24.07 | 20.41 | 16.53 |
| STDEV YOD | 19.45 | 18.25 | 19.40 | 20.29 | 26.86 |
| STDEV YNWD | 11.42 | 22.28 | 19.49 | 5.42 | 9.75 |
| CI LOD | 8.14 | 8.21 | 10.55 | 8.95 | 7.25 |
| CI YOD | 8.53 | 8.00 | 8.50 | 8.89 | 11.77 |
| CI YNWD | 5.13 | 10.02 | 8.76 | 2.44 | 4.38 |
| p-value: YNWD vs LOD | 0.003 | <0.001 | 0.002 | 0.001 | <0.001 |
| p-value: YNWD vs YOD | <0.001 | 0.01 | 0.01 | 0.06 | 0.02 |

| **Presenting complaint** |
| --- |

|  | Memory | Cognitive impairment | Mood | Visuospatial |
| --- | --- | --- | --- | --- |
| LOD | 26.00 | 2.00 | 2.00 | 0.00 |
| YOD | 21.00 | 1.00 | 8.00 | 3.00 |
| YRWD | 14.00 | 14.00 | 2.00 | 0.00 |

**Diagnosis**

|  | Alzheimer's disease | Vascular dementia | Frontotemporal dementia | Dementia secondary to neurological disorder | Unspecified dementia | Anxiety disorder | Alcoholic dementia | Mild cognitive impairment |
| --- | --- | --- | --- | --- | --- | --- | --- | --- |
| LOD | 25.00 | 2.00 | 0.00 | 2.00 | 0.00 | 0.00 | 0.00 | 1.00 |
| YOD | 19.00 | 5.00 | 1.00 | 1.00 | 1.00 | 2.00 | 1.00 | 0.00 |
| YRWD | 6.00 | 1.00 | 0.00 | 1.00 | 0.00 | 7.00 | 1.00 | 19.00 |

| **Time from symptom onset to diagnosis** |
| --- |

|  | < 6 months | < 1 year | < 3 years | < 5 years | > 5 years |
| --- | --- | --- | --- | --- | --- |
| LOD | 3.00 | 6.00 | 11.00 | 6.00 | 3.00 |
| YOD | 3.00 | 4.00 | 7.00 | 3.00 | 1.00 |
| YRWD | 2.00 | 7.00 | 14.00 | 6.00 | 1.00 |

| **Comorbidities** |
| --- |

|  | Diabetes | Cardiovascular | Neurological | Depression |
| --- | --- | --- | --- | --- |
| Late onset dementia | 8.00 | 22.00 | 5.00 | 11.00 |
| Young referral with dementia | 8.00 | 9.00 | 2.00 | 24.00 |
| Young referral without dementia | 6.00 | 11.00 | 9.00 | 21.00 |
| p-value LOD vs YRWD | 0.76 | 0.01 | 0.36 | 0.02 |
| p-value LOD vs YOD | 1 | 0.002 | 0.42 | 0.002 |
| p-value YOD vs YRWD | 0.76 | 0.78 | 0.045 | 0.55 |

| **Medication** |
| --- |

|  | Donepezil | Memantine | Rivastigmine | Galantamine | Antidepressants | Antipsychotics | Benzodiazepines |
| --- | --- | --- | --- | --- | --- | --- | --- |
| Late onset dementia | 8.00 | 4.00 | 5.00 | 2.00 | 9.00 | 2.00 | 1.00 |
| Young onset dementia | 20.00 | 6.00 | 3.00 | 2.00 | 24.00 | 5.00 | 8.00 |
| Young referral | 4.00 | 0.00 | 0.00 | 0.00 | 18.00 | 0.00 | 2.00 |
| p value: YOD vs LOD | 0.0044 | 0.73 | 0.71 | 1 | 0.00028 | 0.42 | 0.026 |

**Statistical tests**

Chi squared (including Yates correction):


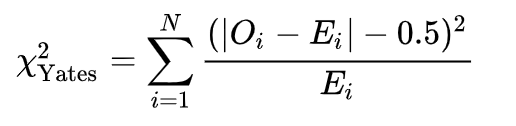


Two-sample unpaired t-test:


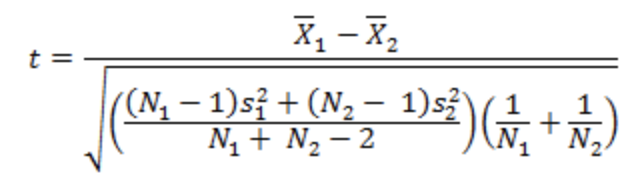


Fisher’s exact test:


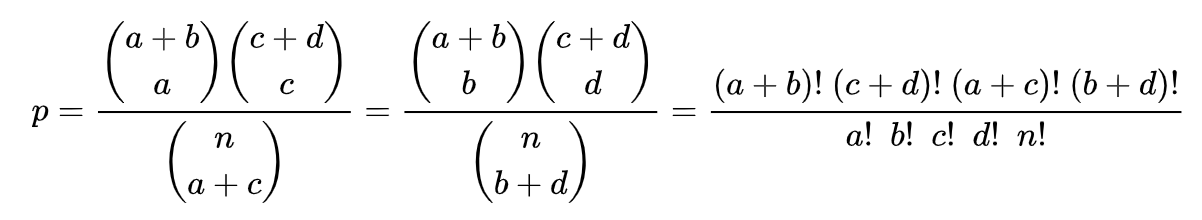

Supplement: Supplementary file 1 [file S2056469420000893sup001.zip › S2056469420000893sup001.docx]
